# Music therapy intervention in cardiac autonomic modulation, anxiety, and depression in mothers of preterms: randomized controlled trial

**DOI:** 10.1186/s40359-018-0271-y

**Published:** 2018-12-13

**Authors:** Mayara K. A. Ribeiro, Tereza R. M. Alcântara-Silva, Jordana C. M. Oliveira, Tamara C. Paula, João B. R. Dutra, Gustavo R. Pedrino, Karina Simões, Romes B. Sousa, Ana C. S. Rebelo

**Affiliations:** 1 School of Medicine, Universidade Federal de Goiás, Goiânia, GO Brazil; 2 School of Music and Performing Arts, Universidade Federal de Goiás, Goiânia, GO Brazil; 3 Center of Neuroscience and Cardiovascular Research, Universidade Federal de Goiás, Goiânia, GO Brazil; 4 Department of Morphology, Biological Sciences Institute, Universidade Federal de Goiás, Goiânia, GO Brazil

**Keywords:** Heart rate variability, Anxiety, Depression, Mothers, Music therapy

## Abstract

**Background:**

Mothers of preterm infants often have symptoms of anxiety and depression, recognized as risk factors for the development of cardiovascular diseases and associated with low rates of heart rate variability (HRV). This study aimed to evaluate the influence of music therapy intervention on the autonomic control of heart rate, anxiety, and depression in mothers.

**Methods:**

Prospective randomized clinical trial including 21 mothers of preterms admitted to the Neonatal Intensive Care Unit of a tertiary hospital, recruited from August 2015 to September 2017, and divided into control group (CG; *n* = 11) and music therapy group (MTG; *n* = 10). Participants underwent anxiety and depression evaluation, as well as measurements of the intervals between consecutive heartbeats or RR intervals for the analysis of HRV at the first and the last weeks of hospitalization of their preterms. Music therapy sessions lasting 30–45 min were individually delivered weekly using receptive techniques. The mean and standard deviation of variables were obtained and the normality of data was analyzed using the Kolmogorov-Smirnov test. The paired sample t-test or Wilcoxon test were employed to calculate the differences between variables before and after music therapy intervention. The correlations anxiety versus heart variables and depression versus heart variables were established using Spearman correlation test. Fisher’s exact test was used to verify the differences between categorical variables. A significance level of *p* < 0.05 was established. Statistical analysis were performed using the Statistical Package for the Social Sciences, version 20.

**Results:**

Participants in MTG had an average of seven sessions of music therapy, and showed improvement in anxiety and depression scores and autonomic indexes of the time domain (*p* < 0.05). Significant correlations were found between depression and parasympathetic modulation using linear (*r* = − 0.687; *p* = 0.028) and nonlinear analyses (*r* = − 0.689; *p* = 0.027) in MTG.

**Conclusion:**

Music therapy had a significant and positive impact on anxiety and depression, acting on prevention of cardiovascular diseases, major threats to modern society.

**Trial registration:**

Brazilian Registry of Clinical Trials (no. RBR-3x7gz8). Retrospectively registered on November 17, 2017.

## Background

Hospitalization of preterm infants in a Neonatal Intensive Care Unit (NICU) can be a time of great suffering for both the family and the patient. Under these circumstances, parents, especially mothers, may experience a number of reactions, including sadness, fear, disappointment, anger, and helplessness [1]. Parents should be encouraged to express any feelings of guilt, anxiety, inadequacy, or anger and also ask for help and/or support. This way, they may be able to better cope with these negative emotions and to understand that these are normal reactions experienced by most parents who face this situation [2].

Anxiety and depression are recognized as significant risk factors for the development of cardiovascular diseases [3, 4], and therefore can compromise the health and well-being of individuals affected by them. They have also been associated with changes in cardiovascular modulation and sympathovagal balance measured by heart rate variability (HRV) indices [5]. Overall, HRV describes oscillations in the intervals between consecutive heartbeats (RR intervals) caused by the influences of the autonomic nervous system (ANS) on the sinus node [6, 7]. Among several methods used to evaluate autonomic modulation, HRV has emerged as a simple, noninvasive measurement technique and has been considered one of the most promising markers of autonomic balance [8].

Taking these risks into consideration, it is important to propose strategies to minimize the symptoms of anxiety and depression. One of the strategies is music therapy, defined by the American Music Therapy Association as the clinical and evidence-based use of musical interventions to meet individualized goals within a therapeutic relationship by an accredited professional who has completed an approved music therapy program [9].

Music therapy interventions, performed with the use of receptive techniques, have been proven to significantly reduce anxiety levels [10]. During kangaroo care, music therapy intervention using the harp had a significant effect to minimize the level of anxiety of mother–baby dyads compared to the control group in a randomized study [11]. Another randomized study with mothers and their infants in a NICU showed that: a) the group in which maternal singing was associated with kangaroo care had a significant reduction in maternal anxiety levels compared to that under kangaroo care without music intervention; b) the preterms exhibited better autonomic stability, with significant change in low frequency (LF) and high frequency (HF) and lower LF/HF ratio, during kangaroo care in association with maternal singing, both during the intervention and recovery phases, compared to those under kangaroo care without music intervention and baseline (*p* = − 0.05) [12].

Studies that evaluate the benefits of music therapy for mothers of preterm infants are still scarce [13], and so are those correlating anxiety and depression with cardiovascular autonomic dysfunction assessed by HRV. Therefore, the present study aimed to evaluate the influence of music therapy intervention on the autonomic control of heart rate, anxiety, and depression in mothers of preterm infants admitted to the NICU. We hypothesized that music therapy is able to reduce the symptoms of anxiety and depression as well as increase HRV in mothers of preterm infants in the NICU.

## Methods

This is a prospective randomized clinical trial that included mothers of preterm infants admitted to the NICU of the Women’s Hospital and Maternity Dona Iris (WHMDI), a tertiary hospital in Goiânia, GO, Brazil, recruited from August 2015 to September 2017. The research project was approved by the WHMDI Academic Board and the Ethics and Research Committee of the Universidade Federal de Goiás (no. 636368). It was registered in the Brazilian Registry of Clinical Trials (no. RBR-3x7gz8) and complies with the principles of the Committee on Publication Ethics.

### Inclusion and exclusion criteria

Mothers (18–40 years old) of preterm infants admitted to the NICU of the WHMDI with prediction of at least one-month hospitalization were included. Exclusion criteria were cognitive alteration and/or auditory deficiency that prevented comprehension of the evaluations and questionnaires involved, uncontrolled systemic diseases, use of beta-blockers or antidepressants, and continued use of illicit drugs and/or alcohol during pregnancy and postpartum.

### Randomization

The determination of the number of volunteer participants was based on a pilot study conducted by our research group. The mean and standard deviation (SD) of root mean square of successive differences between adjacent RR intervals (RMSSD) were calculated. This is a parameter to evaluate parasympathetic modulation, employed in this calculation since it is considered appropriate to cross anxiety and depression data. Sample calculation was carried out using the GPower 3.1.9.2 application for the 95% confidence interval, study power of 80%, and Effect Size d 0.89. Therefore, the sample size was determined as 36 individuals (24 participants in music therapy group – MTG; 12 participants in control group – CG). Considering a possible sample loss during the study, 46 participants were recruited in the first week of admission of their preterms in the NICU and their informed consent was obtained. To carry out simple randomization, 50 kraft sealed envelopes containing the names of the groups (CG and MTG) in identical proportions were used to assign participants to each group. The randomized envelope was opened by the participant or by the researcher within her line of sight, resulting in: 21 participants in CG and 25 participants in MTG. Due to the deadline of the funding institution, it was not possible to randomize 50 participants.

### Evaluation

To evaluate anxiety, depression, and HRV, all the participants responded to the validated Brazilian Portuguese versions of the Beck Anxiety Inventory (BAI) and Beck Depression Inventory (BDI) [14], and RR intervals were recorded for the analysis of HRV, respectively, at two different moments, the first and the last weeks of hospitalization of their preterms. Once the preterm was scheduled to be discharged by the medical staff, the mother underwent the final evaluations. In addition, participants responded to a sociodemographic questionnaire.

### Beck scales

BAI and BDI are 21-item self-report inventories designed to measure the intensity of anxiety and depression, respectively, by assessing symptoms commonly associated with these conditions. A psychologist applied BAI and BDI orally and the participants responded using a 4-point Likert scale, ranging from 0 to 3 (0 = not at all bothered; 3 = severely bothered), to express how bothered they felt by each symptom during the past week. The total scores for both scales range from 0 to 63 points. For BDI, total scores indicate that depression is minimal (from 0 to 11 points), mild (from 12 to 19 points), moderate (from 20 to 35 points), or severe (from 36 to 63 points). For BAI, the cut-off points indicate that anxiety is minimal (from 0 to 10 points), mild (from 11 to 19 points), moderate (from 20 to 30 points), or severe (from 31 to 63 points) [14].

### RR intervals recording and HRV analysis

All participants were evaluated in the afternoon to avoid different physiological responses due to circadian changes. The measurements were carried out in an air-conditioned room, at temperatures ranging from 22 °C to 24 °C and relative humidity between 40 and 60%. Each participant was previously instructed: not to ingest stimulant beverages such as caffeine or alcohol the night before and on the day of testing; not to perform moderate or intense exercises the day before the measurements; to avoid copious meals; and to have a light meal at least 2 h before testing.

RR intervals were recorded at rest, while the participants were seated and breathing normally, over a 12-min period, using a cardiofrequencimeter (Polar® V800, Polar Electro Oy, Kempele, Finland). It is worth emphasizing that, in many studies involving music, RR intervals are recorded during music listening, which was not the procedure adopted in the present study. In both the initial and final evaluations of participants in CG and MTG, RR intervals were recorded in silence. And for the final evaluation of participants in MTG, it was analyzed at least 12 h after the last music therapy session. This approach intended to verify the prolonged effects of the music therapy intervention in MTG.

HRV was analyzed using linear (time and frequency domains) and nonlinear methods. The region presenting the greatest stability in the RR interval time series with 256 consecutive beats was selected for the analyses. Artifacts in the RR interval time series were corrected by deletion, interpolation, and using Kubios HRV [15]. Time domain parameters studied were the standard deviation of NN intervals (interbeat intervals from which artifacts have been removed; SDNN) and RMSSD. SDNN reflects overall HRV, whereas RMSSD is an index of cardiac parasympathetic modulation. For frequency domain parameters, spectral analysis was carried out using fast Fourier transform, applied to a single window, after a linear trend subtraction in previously chosen RR intervals. The spectral components were obtained at LF (0.04–0.15 Hz) and HF (0.15–0.4 Hz), in absolute units (ms^2^), and the normalized units were computed by dividing the absolute power of a given LF or HF component (ms^2^) by the total power, subtracting the very low frequency (VLF: 0.003–0.04 Hz) power, and multiplying this ratio by 100. Since the LF band is modulated by both the sympathetic and the parasympathetic nervous systems and the HF band is correlated with vagal cardiac control, the LF/HF ratio was calculated to determine the sympathovagal balance. The VLF band of 0.003 to 0.04 Hz represents the actions of humoral, vasomotor, and temperature regulation in addition to the activity of the renin-angiotensin-aldosterone system [16].

Nonlinear indices representing parasympathetic modulation and overall HRV variability were instantaneous beat-to-beat variability (SD1) and continuous beat-to-beat variability (SD2), with approximate entropy and sample entropy representing HRV complexity [17].

### Music therapy intervention

A music therapy questionnaire [18] was applied to participants in MTG to collect data on their experience with music and a list of favorite songs. Music therapy intervention began after the conclusion of the initial evaluation stage. The sessions, conducted by professional music therapists, were held once a week, individually, and lasted from 30 to 45 min. The number of sessions differed among participants, since they remained in music therapy for the period of hospitalization of their preterm infants in the NICU, which varied according to their clinical situation.

Each music therapy session consisted of the following steps [18]:

1. Reception: meeting the participant in the NICU or her room and taking her to the office for care;
2. Type I music listening: listening to an instrumental piece, for 2 to 4 min, aiming to provide the participant with a moment for quiet reflection to think of her life and the hospitalization of her preterm in the NICU. Instrumental music was chosen to avoid the influence of lyrics on the musical perception of the participant, considering possible associations with a past event, positive or negative. The selection of type I pieces followed these criteria: a) classical music; b) baroque, classical, or romantic periods; c) tonal; d) with regular pulse; e) containing few points of tension, followed by tension resolution; f) with low levels of dissonance. Predictability, generated mainly by regular pulse, harmonic cadence following a tonal axis, and resolutive endings are important features to provide the listener with a sense of security. Instrumental pieces, usually solos or duets, in slow tempo [60 to 80 beats per minute (bpm)] [19], with clearly delineated musical phrases were chosen for this phase. The same pieces were used in the same sequence for all participants in MTG;
3. Therapeutic music listening: nomenclature proposed by Alcântara-Silva [18] aiming to establish some differences in relation to music listening “in therapy” or “in medicine”. It differs from other studies because the present technique is inserted in the therapeutic context in a processual manner, while in other studies the musical intervention often happens in a single moment [18]. The musical repertoire used in therapeutic music listening consisted of songs selected by the participant, unlike most other studies, in which the researcher selects them;
4. Verbal processing: a moment for the participant to freely share her experience of therapeutic music listening. The purpose of this procedure is to help participants use musical expression to find their own coping strategies, so that they can be strengthened to face moments of anguish and fragility;
5. Type II music listening: the selection of type II pieces followed the same criteria described for type I selection (a–f). However, in this phase the repertoire consisted mainly of densely textured pieces, composed for orchestras, with various timbres, progressing faster than type I pieces (above 80 bpm). All pieces were instrumental, except for the last one, which was vocal. The same pieces were used in the same sequence for all participants in MTG;
6. Conclusion: the music therapist briefly commented the issues approached during that session, set up the date for the following one, and concluded the session.

### Statistical analysis

The mean and SD of each variable were calculated. The normality of data was analyzed using the Kolmogorov-Smirnov test. The differences between the variables evaluated before and after music therapy intervention were calculated using paired sample t-test or Wilcoxon test. The correlations between anxiety and heart variables and between depression and heart variables were established using Spearman correlation test. The differences between categorical variables were calculated using Fisher’s exact test. Effect size measures were calculated dividing the mean difference by its SD at two different moments, the first and the last weeks of hospitalization of the preterms. The magnitude of the effect size was categorized following these criteria: 0.2 < d < 0.5 = small; 0.5 < d < 0.8 = medium; and d > 0.8 = large [20]. A significance level of *p* < 0.05 was established. Statistical analyses were performed using the Statistical Package for Social Sciences, version 20 (Chicago, IL, United States).

## Results

**Fig. 1:**
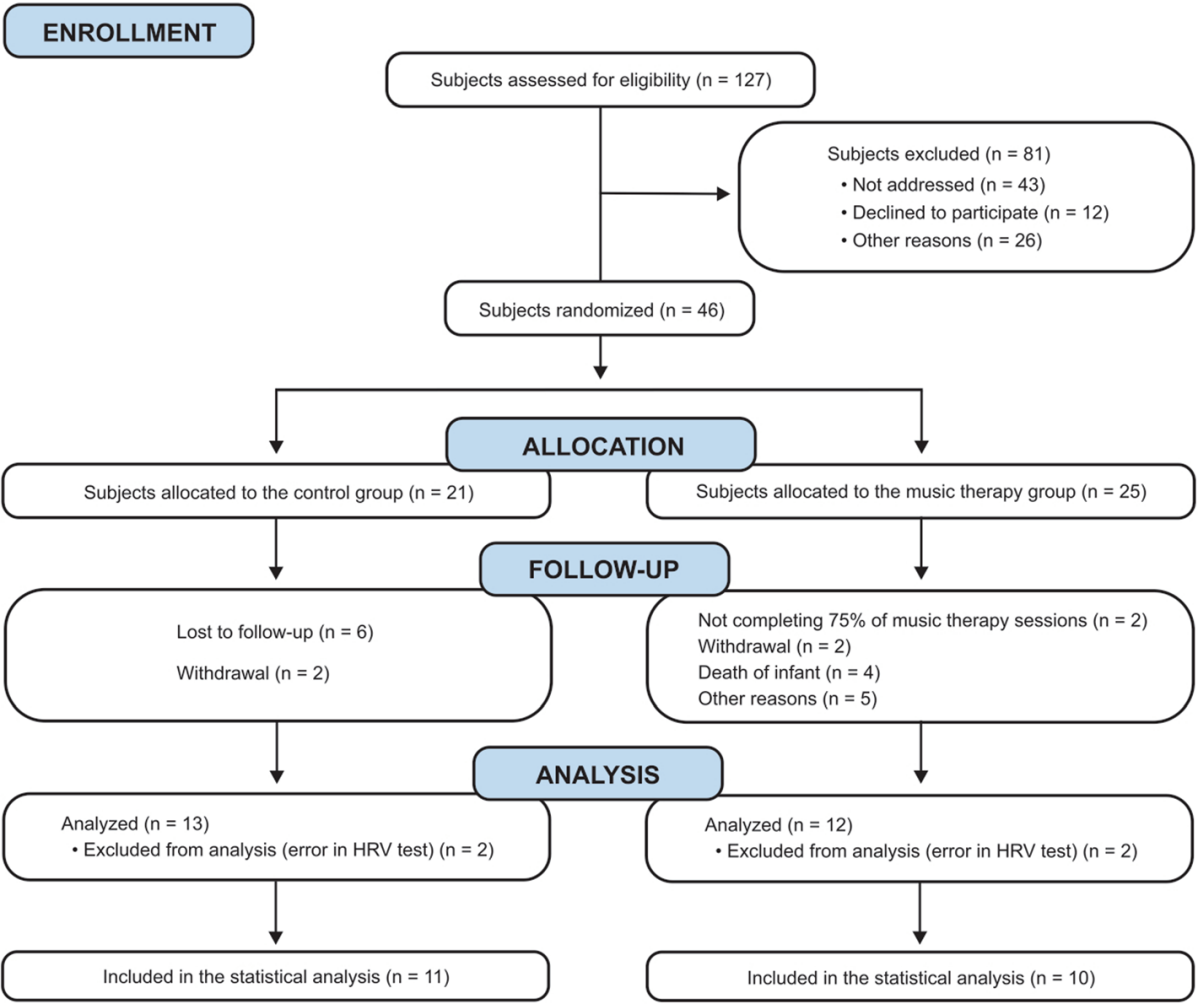
Overview of the study design based on CONSORT diagram

Between August 2015 and September 2017, 46 mothers were recruited and randomly assigned to CG or MTG, as shown in the CONSORT diagram (Fig. 1). In spite of the high number of participants enrolled, data collection was completed for 21 mothers (CG: 11; MTG: 10), not reaching the sample size determined for the study.

Several reasons interfered in the participation of the mothers in this study: a) some babies were discharged before the due date, and because the mothers did not live in the city where the study was performed, they missed the music therapy sessions and/or the final evaluation; b) some mothers had difficulty in staying at the hospital to follow their babies and missed the music therapy sessions and/or the final evaluation; c) non-completion of 75% of music therapy sessions for participants in MTG; d) death of infants (four mothers that participated in MTG lost their babies during the study; although they were offered music therapy support to cope with their grief and mourning, their participation was not included in the statistical analysis to avoid bias); e) withdrawal for personal reasons; f) error in the final HRV test, preventing comparison between the first and the last results.

The sociodemographic profile of the participants is summarized in Table 1. The mean age of the participants at the beginning of the study was 25.8 ± 4.5 years in MTG and 26.2 ± 7.1 years in CG, showing that the sample was homogeneous (*p* = 0.4198). The mean age at first pregnancy was 20.5 ± 3.5 years in MTG and 24.4 ± 7.8 years in CG.

**Table 1:** Sociodemographic profile of the participants in this study

Sociodemographic feature		Music therapy group n (%)	Control group n (%)
Age band (years)	18–23	3 (30)	4 (36.4)
	24–29	3 (30)	4 (36.4)
	30–35	3 (30)	1 (9.1)
	36–40	1 (10)	2 (18.2)
Race	White	5 (50)	4 (36.4)
	Black	1 (10)	0
	Brown	4 (40)	7 (63.6)
Marital status	Married	3 (30)	4 (36.4)
	Single without a partner	1 (10)	1 (9.1)
	Single with a partner	5 (50)	6 (54.5)
	Divorced	1 (10)	0
Family income (minimum wage)	1	4 (40)	2 (18.2)
	2 to 3	5 (50)	6 (54.5)
	Above 3	1 (10)	3 (27.3)
Level of education	Less than primary education	1 (10)	0
	Primary education	1 (10)	1 (9.05)
	Lower secondary education	1 (10)	4 (36.4)
	Upper secondary education	5 (50)	3 (27.3)
	Incomplete tertiary education	1 (10)	2 (18.2)
	Complete tertiary education	1 (10)	1 (9.05)
Occupation	Homemaker	2 (20)	4 (36.4)
	Other	8 (80)	7 (63.6)
Physical activity	Sedentary	6 (60)	8 (72.7)
	Not very active	4 (40)	1 (9.1)
	Active	0	2 (18.2)
Frequency of leisure activities	Once a week	5 (50)	6 (54.5)
	Once a fortnight	1 (10)	3 (27.3)
	Once a month	3 (30)	1 (9.1)
	Rarely or never	1 (10)	1 (9.1)
Religion	Catholic	4 (40)	5 (45.4)
	Protestant	6 (60)	4 (36.4)
	None	0	2 (18.2)
Pregnancy (no.)	1	3 (30)	4 (36.4)
	2	3 (30)	6 (54.5)
	3	2 (20)	0
	4	0	0
	5	2 (20)	1 (9.1)
Age band at first pregnancy (years)	18–23	8 (80)	7 (63.6)
	24–29	2 (20)	4 (36.4)
	30–35	0 (0)	0 (0)
	36–40	0 (0)	0 (0)
Child (no.)	1	7 (70)	6 (54.5)
	2	2 (20)	3 (27.3)
	3	1 (10)	1 (9.1)
	4	0	1 (9.1)
Abortion	Yes	6 (60)	4 (36.4)
	No	4 (40)	7 (63.6)

The mothers allocated to MTG had an average of 7 ± 2 music therapy sessions. The psychological variables anxiety and depression, analyzed using t-test, exhibited significant improvement in MTG, but not in CG (Table 2). They were also investigated using Fisher’s exact test (Table 3) [14], and a migration from higher to lower levels of anxiety and depression was observed in both groups, comparing the outcomes in the initial and final evaluations. However, significant improvement was registered in MTG only for anxiety.

**Table 2:** Psychological and cardiological outcomes in the initial and final evaluations

Parameter	Music therapy group			Control group		
	Initial Mean ± SD	Final Mean ± SD	Effect size	Initial Mean ± SD	Final Mean ± SD	Effect size
BAI	15.10 ± 10.25	5.40 ± 4.72*	0.519 (M)	10.70 ± 8.54	6.00 ± 4.94	0.319 (S)
BDI	15.70 ± 10.68	6.30 ± 5.52*	0.483 (S)	16.00 ± 17.95	10.20 ± 16.26	0.163 (S)
RR intervals	718.80 ± 101.73	630.70 ± 402.50	0.148 (S)	770.53 ± 121.14	745.95 ± 122.88	0.100 (S)
SDNN (ms)	35.01 ± 14.92	44.53 ± 12.95*#	−0.322 (S)	41.35 ± 19.90	43.53 ± 22.11	− 0.051 (S)
RMSSD (ms)	23.66 ± 10.21	36.59 ± 17.58*	− 0.410 (S)	28.14 ± 15.82	31.59 ± 20.07	−0.095 (S)
pNN50 (%)	6.08 ± 7.96	18.37 ± 17.30*	− 0.415 (S)	8.85 ± 12.08	11.54 ± 17.96	− 0.087 (S)
SD1	16.76 ± 7.22	25.91 ± 12.45*	− 0.410 (S)	18.41 ± 12.68	22.41 ± 14.34	− 0.146 (S)
SD2	46.50 ± 20.16	56.95 ± 15.63	− 0.278 (S)	51.75 ± 30.35	58.25 ± 28.32	− 0.110 (S)
DFA α1	1.11 ± 0.22	1.05 ± 0.35	0.102 (S)	1.12 ± 0.24	1.16 ± 0.23	−0.084 (S)
DFA α2	0.94 ± 0.16	0.89 ± 0.24	0.121 (S)	1.05 ± 0.34	0.86 ± 0.21	0.318 (S)
VLF (0–0.04 Hz)	733.50 ± 774.68	823.60 ± 675.78	−0.061 (S)	1039.20 ± 1026.09	943.10 ± 1432.13	0.038 (S)
LF (0.04–0.15 Hz)	508.90 ± 433.67	630.70 ± 402.50	−0.144 (S)	504.50 ± 419.49	601.30 ± 595.61	−0.093 (S)
HF (0.15–0.4 Hz)	565.20 ± 1013.54	611.10 ± 462.50	−0.029 (S)	397.30 ± 331.67	435.60 ± 503.28	−0.044 (S)
Total	1808.00 ± 1798.51	1950.12 ± 1364.49	−0.044 (S)	1941.20 ± 1660.41	1980.00 ± 2200.50	−0.009 (S)
LF/HF	1.95 ± 1.14	1.85 ± 2.01	0.030 (S)	2.20 ± 2.23	1.88 ± 1.23	0.088 (S)
LF (n.u.)	60.14 ± 18.61	53.35 ± 21.43	0.166 (S)	58.38 ± 19.23	60.13 ± 14.35	−0.051 (S)
HF (n.u.)	39.83 ± 18.62	46.65 ± 21.45	− 0.167 (S)	41.60 ± 19.26	39.87 ± 14.35	0.050 (S)

*SD* Standard deviation, *BAI* Beck Anxiety Inventory, *BDI* Beck Depression Inventory, *RR intervals* Intervals between consecutive heartbeats, *SDNN* Standard deviation of NN intervals, *NN intervals* Interbeat intervals from which artifacts have been removed, *RMSSD* Root mean square of successive differences between adjacent RR intervals, *pNN50*, NN50 count divided by the total number of NN intervals, *NN50* Number of successive NN intervals differing more than 50 ms, *SD1* Instantaneous beat-to-beat variability, *SD2* Continuous beat-to-beat variability, *DFA α1* Detrended fluctuation analysis of short-term fractal scaling exponents, *DFA α2* Detrended fluctuation analysis of long-term fractal scaling exponents, *VLF* Very low frequency, *LF* Low frequency, *HF* High frequency, *(M)* Medium effect size [20], *(S)* Small effect size [20]; *significant at *p* ≤ 0.05 in intergroup evaluation in the final evaluation using paired sample t-test or Wilcoxon test; # significant at *p* < 0.05 in intergroup evaluation in the final evaluation using t-test

**Table 3:** BAI and BDI scores in the initial and final evaluations

Scale	Score	Music therapy group			Control group		
		Initialn (%)	Final n (%)	*p*	Initial n (%)	Final n (%)	*p*
BAI	Minimum	4 (40)	9 (90)	0.045*	7 (63.6)	9 (81.8)	0.522
	Light	2 (20)	1 (10)		2 (18.2)	2 (18.2)	
	Moderate	4 (40)	0		2 (18.2)	0	
	Serious	0	0		0	0	
BDI	Minimum	4 (40)	7 (70)	0.150	6 (54.5)	8 (72.7)	0.747
	Light	2 (20)	3 (30)		2 (18.2)	2 (18.2)	
	Moderate	4 (40)	0		2 (18.2)	0	
	Serious	0	0		1 (9.1)	0	

*BAI* Beck Anxiety Inventory, *BDI* Beck Depression Inventory; *significant at *p* ≤ 0.05 using Fisher’s exact test

Comparisons between the groups showed that time domain parameters (SDNN, RMSSD, and pNN50) and nonlinear dynamics (SD1 index) presented a lower mean value in MTG compared to CG in the initial evaluation. This scenario reversed after the music therapy intervention, and a significant increase in these parameters was found for participants in MTG, who had higher values than those observed for the participants in CG (Table 2). No significant changes in frequency domain parameters were registered for either group.

**Fig. 2:**
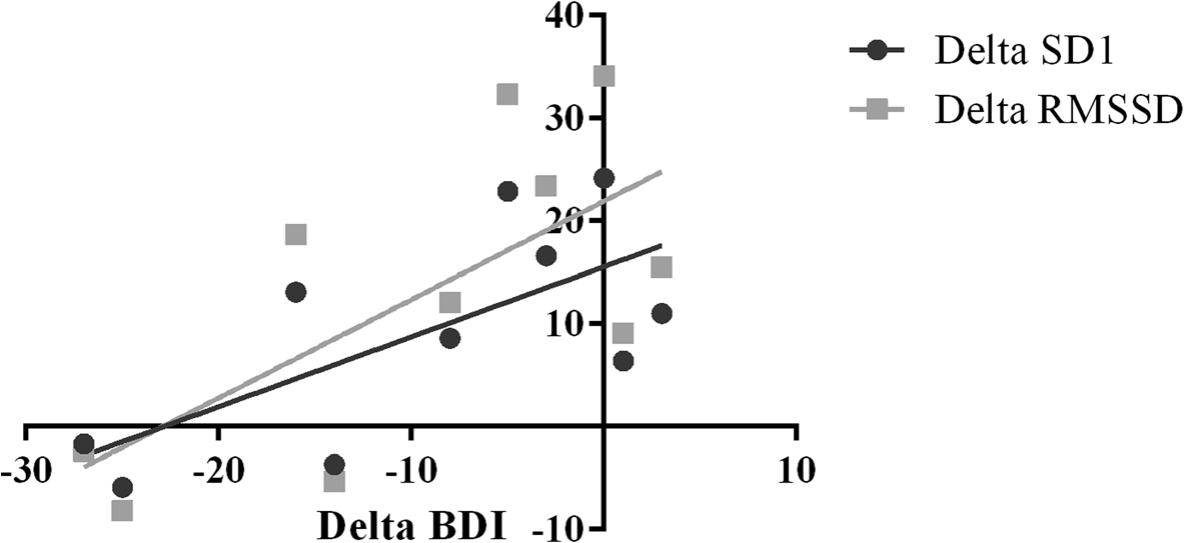
Correlation between the deltas of the Beck Depression Inventory results and the deltas of the heart rate variability indices (RMSSD and SD1). BDI: Beck Depression Inventory; SD1: instantaneous beat-to-beat variability; RMSSD: root mean square of successive differences between adjacent RR intervals (ms).

After the music therapy sessions, significant correlations were found between BDI and RMSSD (*r* = − 0.687; *p* = 0.028) and SD1 (*r* = − 0.689; *p* = 0.027) for participants in MTG, using Spearman correlation test, demonstrating an inversely proportional correlation between HRV and the clinical symptomatology of depression (Fig. 2). Despite this trend, no correlations were observed between BAI scores and psychophysiological variables based on HRV analysis.

## Discussion

It is already well known that music therapy decreases the levels of anxiety and depression in different clinical contexts [3, 21, 22]. Nonetheless, studies including preterm mothers have predominantly addressed the beneficial effects of music therapy only on anxiety scores, not approaching depression scores [11, 23].

In this study, therapeutic music listening was adopted as the therapeutic procedure, i.e. the participants listened to familiar songs selected by themselves, and the sessions took place once a week individually with each mother, without the presence of the preterm, to offer specific therapeutic support to her needs. Familiar songs can help control anxiety, improve concentration, recover memories, provide a sense of security and motivation, and stimulate social interaction, simultaneously giving people the opportunity to recognize and improve their emotions [24]. In fact, participants in MTG were able to express their feelings about their preterm infants or any other situations that were causing them distress or discontent. In other studies, music therapy sessions were intended to improve mother–baby relationship [11, 25, 26], with no specific concern for maternal health.

The present study demonstrated statistically significant improvements on both anxiety and depression scores in MTG. This finding confirmed our hypothesis that the use of music therapy can reduce the symptoms of anxiety and depression in mothers of preterm infants in the NICU. However, the improvement in depression raw scores did not necessarily have an impact on the level of depression as determined by BDI (minimum, mild, moderate, or severe).

Several other studies have shown improvement in depressive and anxious states as a result of music therapy interventions [18, 27]. The beneficial effects on the symptoms of anxiety and depression found in this study corroborate the neurophysiological basis of listening to familiar songs. Listening to pleasant music promotes emotional self-regulation [28] by increasing dopaminergic activity [29, 30] in the ventral striatum and ventral tegmental area and by decreasing the reactivity of the hypothalamic-pituitary-adrenal axis. In turn, these changes decrease serum cortisol levels [31], increase the synthesis and release of central and peripheral endocannabinoids such as anandamide and endorphins, and increase the predominance of parasympathetic heart modulation [32].

HRV results (SDNN, rMSSD, LF, and HF) in the first evaluation were not within the normal range [7] in the sample studied. Stress and anxiety related to having their children hospitalized, as well as the high degree of sedentarism of mothers in both groups (60% in MTG and 72.7% in CG, Table 1) may justify these findings.

Poincaré plot indices SD1 and SD2 indicated similar results, but this method has the advantages of easier calculation and lower stationarity dependence. Indeed, according to these results, SD1 was higher in participants in MTG after music therapy intervention. As demonstrated by our findings, time domain analysis and SD1, both reflecting parasympathetic modulation, mainly identified differences between individuals before and after music therapy intervention.

Music is known to provide a state of relaxation, leading to a reduction in cardiac function in rest periods due to the elevation of parasympathetic modulation [33]. This reduction generates better electrical stability of the heart by decreasing the heart rate, the force of contraction of the atrial muscle, the conduction velocity of cardiac impulse in the atrioventricular node, and the blood flow through the coronary vessels, as well as by increasing the delay between atrial and ventricular contractions. This state of rest keeps the heart muscle healthy and prevents wear and tear of the organ [34]. Therefore, music therapy provides better electrical stability of the heart.

Neuroanatomical findings point to a connection between descending projections of the lateral hypothalamus and the dorsal motor nucleus of the vagus nerve. The lateral hypothalamus is a limbic structure involved in processing positive emotions and motivation [35, 36]. Thus, it is possible to infer that positive emotions originated during the music therapy intervention in this study sensitized the lateral hypothalamus of the participants and, consequently, maximized the vagal action on the heart, contributing to increased parasympathetic modulation.

Another factor that supports the predominance of parasympathetic modulation is that listening to familiar songs can stimulate the central and peripheral production and release of nitric oxide (NO) [32, 35]. Among the many other biological roles played by NO, it acts on the peripheral vasomotor tone, characterized by vasodilation and reduction of blood pressure values. For this reason, the action of NO on the cardiovascular system is one of the ways to explain the parasympathetic predominance of cardiac autonomic modulation after music therapy intervention.

The cardiovascular system is also sensitive to a wide variety of psychological and behavioral states. In this regard, a decrease in the release of catecholamines (adrenaline and noradrenaline) due to musical stimuli could explain the regulation of cardiovascular variables [37]. In addition, parasympathetic activity predominates during relaxation [38]. Taking into account the decrease in anxiety and depression symptoms after music therapy sessions, it can be inferred that the increase in parasympathetic activity is associated with a positive emotional state. Such inference can be corroborated by the correlation found between depression and HRV indices (SD1 and RMSSD).

The vagus nerve, one of the main elements of the parasympathetic portion of the ANS, represents an important afferent component that directly connects the regions of the brain associated with emotions such as the hypothalamus and amygdala [39], and also controls the concentration of neurotransmitters [40]. Vagal stimulation has been studied for the treatment of depressive disorders [40, 41]. Thus, it is possible that music therapy benefitted the participants in many different ways (anxiety, depression, and cardiovascular aspects) due to the interactions between neurotransmitters and ANS.

In a study population consisting of subjects in good general health, the effects of improvisational music therapy on HRV were evaluated at three different moments, totalling 90 min: 30 min before the music therapy session, 30 min during the session, and 30 min after the session. The deviation of the RR intervals was similar before the beginning and after the end of the music therapy session [42]. Corroborating this outcome, in the present study, no differences were observed between the initial and final evaluations of RR intervals in either group.

In the one hand, in a randomized study using receptive music therapy [43], the same method applied in the present study, HRV was assessed during musical listening, and a significant increase in RR intervals was observed. On the other hand, in our study, this was not found. Therefore, based on this discrepancy of results and due to the scarcity of reference materials, we suggest new studies with a greater number of subjects, as proposed in the initial sample calculation, and HRV evaluation during and after music therapy sessions.

In many studies, HRV has been evaluated under resting or post-exercise recovery conditions, and in most previous studies involving music this parameter has been verified during musical listening [42, 44]. The novelty of the method used in the present research lies in the fact that the final HRV was analyzed at least 12 h after music therapy sessions were concluded, thus allowing us to verify non-immediate benefits of music listening. Given this time lapse, the benefits of music therapy on HRV seem to be prolonged.

Having lost almost 46% of the sample for several reasons was a major setback for our study. Another limitation was the collection of HRV only during rest and not both under rest and under stress, although the former has been well documented in the literature. It is also worth noting the impossibility of carrying out a neuroendocrine evaluation (cortisol and catecholamines) of the participants to confirm the autonomic findings, since the appropriate control of their diet was not feasible in a hospital setting.

We hope that our results stimulate future studies that corroborate the influence of music therapy on the physical and emotional well-being of mothers whose preterm infants are in the NICU. It would also be important to conduct studies encompassing other types of population aiming to evaluate the potential of music therapy for cardiac rehabilitation and psychophysiological improvement.

## Conclusion

Anxiety, depression, and HRV were analyzed in mothers of preterms admitted to the NICU before and after music therapy sessions to evaluate the effects of this type of intervention. To our knowledge, no similar studies have been conducted. Parasympathetic activity increased after music therapy sessions, which suggests that music listening can reduce anxiety and depression under the conditions tested. Therefore, it can be considered a reliable and low-cost therapy to be adopted by public health systems. The effect of music therapy on cardiac autonomic modulation provides preliminary clinical evidence of its use as a strategy for cardiovascular disease prevention.

## Abbreviations

ANS: Autonomic nervous system
BAI: Beck anxiety inventory
BDI: Beck depression inventory
bpm: Beats per minute
CG: Control group
HF: High frequency
HRV: Heart rate variability
LF: Low frequency
MTG: Music therapy group
NICU: Neonatal intensive care unit
NN intervals: Interbeat intervals from which artifacts have been removed
NN50: Number of successive NN intervals differing more than 50 ms
NO: Nitric oxide
pNN50: NN50 count divided by the total number of NN intervals
RMSSD: Root mean square of successive differences between adjacent RR intervals;
RR intervals: Intervals between consecutive heartbeats
SD1: Instantaneous beat-to-beat variability
SD2: Continuous beat-to-beat variability
SDNN: Standard deviation of NN intervals
VLF: Very low frequency
WHMDI: Women’s Hospital and Maternity Dona Iris.
